# Attenuation of High-Frequency (50-200 Hz) Thalamocortical EEG Rhythms by Propofol in Rats Is More Pronounced for the Thalamus than for the Cortex

**DOI:** 10.1371/journal.pone.0123287

**Published:** 2015-04-15

**Authors:** Sean J. Reed, Gilles Plourde

**Affiliations:** 1 Integrated Program in Neuroscience, McGill University, Montréal, Canada; 2 Department of Anesthesia, McGill University, Montréal, Canada; University of British Columbia, CANADA

## Abstract

**Background:**

Thalamocortical EEG rhythms in gamma (30-80 Hz) and high-gamma (80-200 Hz) ranges have been linked to arousal and conscious processes. To test the hypothesis that general anesthetics attenuate these rhythms, we characterized the concentration-effect relationship of propofol on the spectral power of these rhythms. In view of the ongoing debate about cortex versus thalamus as the primary site of anesthetic action for unconsciousness, we also compared the relative sensitivity of cortex and thalamus to this effect propofol.

**Methods:**

Adult male Long-Evans rats were chronically implanted with electrodes in somatosensory (barrel) cortex and ventroposteromedial thalamus. Propofol was delivered by a computer-controlled infusion using real-time pharmacokinetic modeling to obtain the desired plasma concentration. Spectral power was assessed during baseline, at four stable propofol plasma-concentrations (0, 3,6,9,12 μg/ml) and during recovery over four frequency ranges (30-50, 51-75, 76-125, 126-200 Hz). Unconsciousness was defined as complete loss of righting reflex. Multiple regression was used to model the change of power (after logarithmic transformation) as a function of propofol concentration and recording site.

**Results:**

Unconsciousness occurred at the 9 μg/ml concentration in all animals. Propofol caused a robust linear concentration-dependent attenuation of cortical power in the 76-200 Hz range and of thalamic power in the 51-200 Hz range. In all instances the concentration-effect slope for the thalamus was markedly steeper than for the cortex. Furthermore the lowest concentration causing unconsciousness significantly reduced cortical power in the 126-200 Hz range and thalamic power in the 51-200 Hz range.

**Conclusions:**

Propofol causes a concentration-dependent attenuation of the power of thalamocortical rhythms in the 51-200 Hz range and this effect is far more pronounced for the thalamus, where the attenuation provides a robust correlate of the hypnotic action of propofol.

## Introduction

The mechanisms by which general anesthetics suppress consciousness remain elusive [1, 2]. The hypothesis that general anesthetics impair consciousness through, in part, attenuation of high-frequency thalamocortical EEG rhythms in the gamma (30–80 Hz) range has been proposed [3–6]. Gamma rhythms are an important element of on-going activity of the waking brain, reflecting the depolarization of thalamic and cortical neurons [7]. They occur in all sensory areas and have been associated with a number of high-level neurocognitive processes including general arousal, sensory, and attention [8, 9]. Early observations of high-frequency rhythms were confined to the gamma range but recent developments have drawn attention to the broadband high-gamma (80–200 Hz) range [10, 11].

There is emerging evidence that anesthetics such as isoflurane and propofol attenuate high-frequency cortical rhythms above 50 Hz in humans and animal models [12–14] as well as thalamocortical network dynamics [15, 16]. We recently reported [17] that propofol anesthesia in rats is associated with a decrease of 80–200 Hz power in the cortex and thalamus and that physostigmine given during continuous infusion of propofol causes behavioral arousal that correlates with an increase in 80–200 Hz power in the thalamus. Furthermore, there was a suggestion in this study that the attenuation of power in the 50–200 Hz range during propofol was more pronounced for the thalamus than for the cortex but the evidence was not sufficiently strong to warrant an explicit statement. Thus further evaluation of the effect of propofol on gamma and high-gamma rhythms on cortex and thalamus appears warranted. Demonstration of a concentration-effect relationship is the standard method to assess the effect of a drug and to provide evidence of causality. A formal comparison of the cortex and thalamus in regard to their sensitivity to propofol could inform the issue of whether anesthetic-induced unconsciousness primarily results for impairment of the cortex, thalamus or both [1].

Our primary aim was to characterize the concentration-effect relationship of propofol for the attenuation of high frequency (30–200 Hz) thalamocortical EEG rhythms and to compare the cortex and thalamus in regard to their sensitivity to propofol. The 30–200 Hz range was divided in the following bands chosen on the basis of prior studies [12, 14]: 30–50 Hz; 51–75 Hz; 76–125 Hz and 126–200 Hz. For each frequency band, we evaluated to concentration effect relationship of propofol on the power of the EEG recorded from the somatosensory (barrel) cortex and ventroposteromedial thalamic nucleus (VPM) of the rats with chronically implanted electrodes and we compared the steepness of the cortical and thalamic regression lines. We chose the ventroposteromedial thalamic nucleus (VPM) because of its documented sensitivity to general anesthetics [18] and because it is suitable for recording spontaneous fast oscillations [19]. The barrel cortex was selected because it receives inputs from the VPM and also displays fast spontaneous oscillation [20, 21].

Our secondary aim was to determine whether the attenuation of high-frequency power in the cortex or thalamus could reflect neural events that play a role in causing unconsciousness. We used the following criterion as a necessary (but not sufficient) condition: if the attenuation of power with a frequency band is linked in any way to unconsciousness, then it should be possible to demonstrate that the highest level of power for this frequency band observed during unconsciousness (i.e. the 9 and 12 μg/ml concentrations) is significantly less than the lowest level of power observed during any period when the animal is conscious. Any attenuation of power that fulfills this stringent criterion can be considered a potential correlate of the hypnotic effect of propofol and would deserve further investigation.

## Materials and Methods

### Ethics Statement

This study was carried out in strict accordance with the guidelines of the Canadian Council on Animal Care. The protocol was approved by the Montreal Neurological Institute Animal Care Committee. All surgery was performed under general anesthesia with ketamine and xylazine. All efforts were made to minimize suffering. Male Long-Evans rats (300–320g, n = 9) were acquired from Charles River Laboratories (Senneville, Quebec), and arrived with a right jugular vein catheter. Animals were housed individually in an enriched environment with food and water available, *ad libitum*. A normal light-cycle (light on from 07:00 to 19:00 Hrs) was maintained throughout the duration of the experiment. After completion of the experiments, the animals were euthanized under deep urethane anesthesia by perfusion via the ascending aorta.

### Surgery

Rats were anesthetized with ketamine (50 mg/kg, ip) / xylazine (5 mg/kg, ip) and secured to a stereotaxic frame which was equipped with a heating pad and rectal temperature monitor. A midline incision was made along the scalp and the periosteum was retracted with hemostats. Bipolar electrodes made of twisted, teflon-coated stainless-steel wires (125μm diameter; vertical separation between the wire tips: 0.5–1.0 mm; AM Systems, WA) were then positioned in the VPM (Paxinos atlas [22] coordinates: A/P: -3.5, L/M: 2.7, V/D: -6.6, relative to bregma) and sensory (barrel) cortex (A/P: -2.3, L/M: 5.0, V/D: -3.6) for local field potentials (LFPs) recordings. LFPs consist of EEG activity recorded with micro-electrodes inserted in brain tissue [23]. The cortical electrode was adjusted vertically to optimize the amplitude of the field excitatory post-synaptic potentials evoked by VPM stimulation. Thicker wires (0.011μm exposed tips, AM Systems, WA) soldered to stainless steel screws were placed in the contralateral parietal bone and the ipsilateral frontal bone to serve as the reference and ground electrodes, respectively. Electrode leads were then fastened to gold-plated Amphenol pins and inserted into a nine-pin ABS connector (Ginder Scientific, Nepean ON, Canada). This assembly was then secured to the skull using acrylic dental cement. All animals were administered ketoprofen (5 mg/kg S.C. at end of surgery and repeated once a day for 2 days) and buprenorphine (0.5 mg/kg S.C. at end of surgery and repeated every 12 hours for up to 3 days if needed). Animals were routinely monitored to minimize any suffering or post-operative complications. One week was allotted for recovery and observation, whilst the catheter was flushed every 3–5 days with a gentamycin / heparin solution. No animals exhibited clinical endpoints warranting removal from the study, including significant weight loss, behavior changes, or other adverse reactions to anesthesia and surgery.

### Design

The experimental sessions were preceded by a forty-minute acclimation session, which occurred daily for three days, allowing the animals to explore the testing chamber. During this time, the animal’s head assembly was attached to the commutator, but with no recording. This reduced any anxiety-provoked excitement that would confound the results.

Each rat underwent a single testing session with six conditions (Baseline—target plasma propofol concentrations of 3 μg /ml, 6 μg/ml, 9 μg/ml, 12.0 μg/ml, and recovery after return of spontaneous ambulation). The choice of these concentrations was based on previous experiments [17] and designed to cover both the sub-hypnotic (3 and 6 μg/ml) and hypnotic (9 and 12 μg/ml) ranges. Our previous work [17] had shown that unconsciousness (defined as loss of righting reflex and absence of any attempts at righting) occurred at concentrations between 6.5 and 9 μg/ml. The initial baseline condition consisted of recording LFPs and observing behavior (ambulation, grooming…) while the rat explored the environment. This sequence was repeated for the 3, 6, 9, and 12 μg/ml concentrations. The animals were closely monitored during the step-wise administration of propofol to detect any unintended effects. Finally, the propofol administration was terminated and the animals were given time to regain their righting reflex and spontaneous ambulation before the last recording (recovery).

The anesthetic was administered in the right jugular vein catheter with a Harvard-22 syringe pump controlled by the Stanpump software developed by Steven L. Shafer and colleagues (Department of Anesthesiology, Standford University, CA) using pharmacokinetic parameters derived from Knibbe et al. [24]. After a 15 minutes drug equilibration period, LFPs were recorded and spontaneous behavior was observed. If the animal was not ambulating, the righting reflex was tested by placing the animal on its side and by observing attempts to right. The animal was considered unconscious if no righting attempt was made.

### Electrophysiological Recording

Local field potentials from the cortical and thalamic contacts were recorded with a common reference on the contralateral parietal bone (referential montage), amplified (0.1 Hz to 475 Hz band pass), digitized at 3030 Hz, and stored for offline analysis. For each period, two minutes of high quality data devoid of artifacts was obtained. The recordings were subsequently reformatted offline to obtain a bipolar recording for each recording site (S1Bf and VPM) consisting of the difference between the recordings of the two contacts (spaced 1 mm from each other) of each electrode. All analyses were conducted with the bipolar recordings because they are less subject to contamination from volume-conducted myogenic artifacts.

Spectral power was computed with the Welch’s method [25] using 2-second long non-overlapping segments and a Hamming window (Matlab Signal Processing toolbox, version 6, MathWorks Inc. Sherborn, MA). Segments containing more than 5% outliers (defined as values outside mean ± three standard deviations of the entire recording of about 2 minutes) were excluded from analysis. The frequency bands were defined as: 30–50, 51–75, 76–125, 126–200 Hz. To minimize the impact of interference from external electrical sources, power at 59–61 Hz, 119–121 Hz, and 179–181 Hz were excluded from analysis. Since baseline power differs between animals, normalization of the spectra is required to avoid an undue influence of animals with high baseline power. The power spectra were normalized for each animal and recording site by dividing the power values by the average power from 0.1–200 Hz during baseline. Thus, for each animal and recording site, the average power during baseline was equal to unity and the spectra for the other periods were scaled with the same parameter.

### Perfusion and Histology

The animals were euthanized under deep urethane anesthesia (2000 mg/kg, I.P) by perfusion via the ascending aorta of 200 ml of heparinized saline followed by 500 ml of 10% neutral buffered formalin. The isolated brains were post-fixed in 4% paraformaldehyde followed by a paraffin embedding. Histological preparations for the first five rats yielded tissue unsuitable for microscopy. The other four brains were processed by the Goodman Cancer Research Centre’s Histology Facility at McGill University. Slices were made in 50 μm step-sections at a thickness of 6 μm and stained with haematoxylin and eosin.

### Statistical Analysis

All power values were transformed with log base 10. Multiple regression was used to model the changes of log power as a linear function of concentration with a common slope for all animals. The recovery period was excluded from regression analysis. A separate analysis was conducted for each frequency band (30–50, 51–75, 76–125, 126–200 Hz). For each analysis, there were 5 repeated measurements (corresponding to the five 5 propofol concentrations; x_j_, j = 1, 2, 3, 4, 5 corresponding to 0, 3, 6, 9, 12 μg/ml) for each recording site (k = 1for cortex and k = 2 for thalamus).

We modeled each outcome y_ijk_ as: < y_ijk_ = α_ik_ + β_0k_ + β_1k_x_j_ + ε_ijk_ > where y_ijk_ denotes the spectral power of rat i (i = 1,2,…,9) at concentration j for site k; α_ik_ is the difference between the random intercept of rat i and β_0k_; β_0k_ is the regression constant for site k; β_1k_ is the regression coefficient for the linear term (i.e. slope) for site k; ε_ijk_ is the error term, which accounts for unexplained errors (e.g., measurement error).

The model contains both fixed effects (the influence of concentration and recording site) and a random effect (the baseline values of each rat). This mixed model was estimated with PROC MIXED in SAS Statistical Software (version 9.2, SAS Institute Inc., Cary, NC) using an autoregressive covariance structure. The choice of a mixed, more complex regression model is required because the experimental design involves serial (repeated) measures in then same animals (see reference [26] for a detailed account).

Inspection of the residuals revealed no anomalies. The p values for the linear term were corrected for multiple comparisons (8 tests) with Hommel's procedure [27]. The goodness of fit was assessed with the concordance correlation coefficient (CCC), which was developed for mixed models as an alternative to the traditional R^2^ in linear regression [28, 29]. This analysis was done for each site separately. The value of CCC ranges from -1 to 1 with a value of -1 reflecting a perfect discordance (similar to a correlation coefficient of -1), a value of 1, perfect concordance and a value of 0, no relationship at all. In the present case we are only interested in the 0 to 1 range.

We compared the cortical and thalamic regression coefficients for the linear term (β_1_) for each frequency range to assess the significance of differences in the slope to concentration-effect plots by modeling signals from two sites simultaneously based on approaches for multivariate longitudinal data [30]. We tested the null hypothesis that (β_1Cortex_—β_1Thalamus_) = 0 assuming that (β_1Cortex_—β_1Thalamus_) / SE (β_1Cortex_—β_1Thalamus_) follows a normal distribution and where SE (β_1Cortex_—β_1Thalamus_) is the standard error of the difference of the coefficients. (We use the underline (instead of the caret (^)) to denote the estimate of a parameter e.g. β_1Cortex_ is the estimate, derived from the data, of β_1Cortex_.) To obtain the standard error, we used the variance of (β_1Cortex_—β_1Thalamus_) computed with the standard formula [31] s_u-v_^2^ = s_u_^2^ + s_v_^2^–2 • r • s_u_ • s_v_ where s_u-v_^2^ is the variance of the difference between the variables u and v; s_u_ and s_v_, their standard deviation and r the correlation coefficient between u and v. These tests were adjusted for multiple comparisons (4 tests) with Hommel’s procedure [27] implemented in SAS Statistical Software (version 9.2, SAS Institute Inc., Cary, NC).

To identify power changes that could potentially be linked to loss of consciousness, we used paired t-tests to compare the concentration with highest mean spectral power (estimated from the entire LFP epoch of 2 minutes and averaged across animals) observed during unconsciousness (i.e. the 9 and 12 μg/ml concentrations) with the period (baseline, 3 or 6 μg/ml concentrations or recovery) with the lowest mean level of power observed when the animal is conscious. This was done for each frequency band and recording site separately, with adjustment for multiple comparisons (8 tests). Paired t-tests were also used to assess the difference in power between the 12 μg/ml concentration and recovery (transition from anesthetized to awake) and were similarly corrected for multiple comparisons (8 tests). Criterion for significance was p≤0.05 with the two-tailed hypothesis.

## Results

### Behavioral Observations

Propofol had a consistent effect on behavior. At the 3 μg/ml concentration, the animals were lethargic with reduced ambulation compared with baseline. The 6 μg/ml concentration was associated with increased ambulation compared with baseline combined with frequent rearings and occasional jumps. The 9 μg/ml abolished righting attempts in all animals, which remained anesthetized until the termination of the propofol infusion. The animals regained consciousness with spontaneous ambulation 8–24 minutes (mean 14.8; std 6.1) after the termination of the propofol infusion.

### Concentration-effect relationships

**Fig 1:**
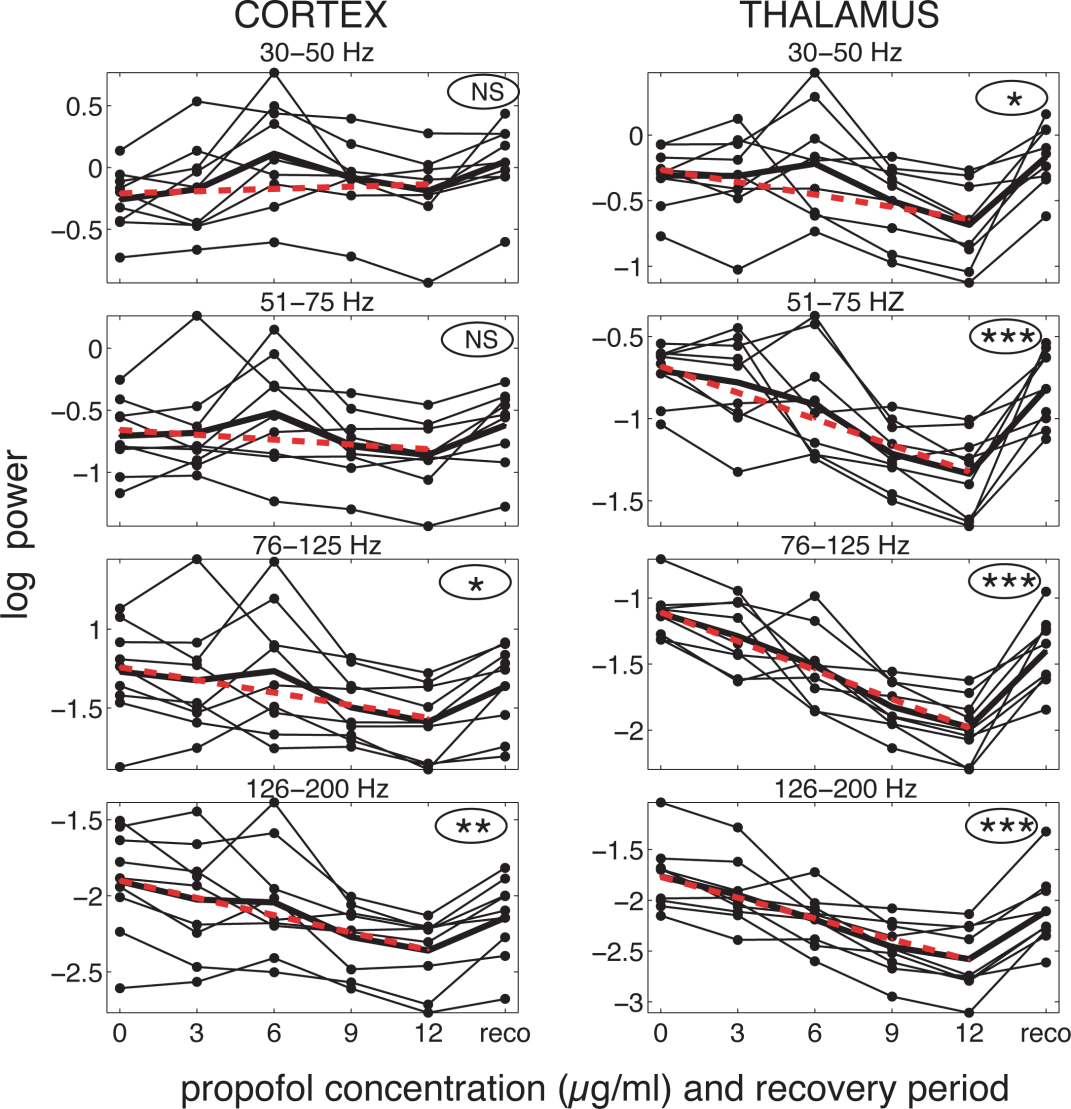
Power as a function of propofol concentration for each frequency band for the cortex and thalamus. Each animal represented by a thin line. The thick black line shows the mean. The dash red line shows the regression fit. Recovery period was not included in the regression. The significance of the regression model is shown in the ellipses: NS: non-significant; *: p<0.05; **: p<0.01; ***: p<0.001; see Table 1 for exact p values and regression parameters estimates.

Fig 1 shows that propofol caused a significant, linear decrease of logarithmic power as a function of propofol concentration for the cortex in the 76–125 Hz and 125–200 Hz frequency ranges as well as for the thalamus in all frequency ranges. More importantly, the slope of the regression line (β_1_ parameter in Table 1) for the thalamus is significantly steeper than for the cortex in all frequency ranges (Table 1). This was a robust effect with p values of 0.0018 for the 126–200 Hz ranges at p<0.0001 for the other frequency ranges. It should be noted that comparisons between slopes for power in the 30–50 Hz and 51–75 Hz ranges are valid even if cortical power in these ranges did not change as a function of propofol concentration (i.e. the slope of the regression line for cortical power did not differ from zero).

**Table 1:** Regression analysis.

	CCC	β_1_	SE β_1_	p β_1_	p adj β1
Cortex					
30–50 Hz	0.05	0.0061	0.0132	0.6443	0.6443
51–75 Hz	0.19	-0.0129	0.0125	0.3041	0.6082
76–125 Hz	0.60	-0.0267	0.0107	***0*.*0147***	***0*.*0441***
126–200 Hz	0.82	-0.0380	0.0100	***0*.*0003***	***0*.*0015***
Thalamus					
30–50 Hz	0.52	-0.0314#	0.0125	***0*.*0145***	***0*.*0435***
51–75 Hz	0.86	-0.0534#	0.0098	***<0*.*0001***	***<0*.*0006***
76–125 Hz	0.51	-0.0729#	0.0089	***<0*.*0001***	***<0*.*0006***
126–200 Hz	0.94	-0.0681&	0.0094	***<0*.*0001***	***<0*.*0006***

CCC: concordance correlation coefficient; SE: standard error of the regression coefficients; p: probability that the regression coefficient is different from zero (based on t-distribution); p adj: p values after correction for multiple (8) comparisons (Hommel's procedure);β_1_: regression coefficient for the linear term (slope); # linear term for thalamus significantly less than for cortex (p<0.0001); & linear term for thalamus significantly less than for cortex (p<0.0018); p values of 0.05 or less are in ***bold italics***.

Table 1 presents in detail the results of the regression analysis including the regression coefficients for the linear term (β_1_, i.e. the slope of the regression line), along with their standard error and the probability that they differ from zero, as well as the concordance correlation coefficient (CCC), which provides a measure of how well the regression parameters fit the data. As expected when the regression analysis yields negative results, the CCCs are low (<0.2) for cortical power in the 30–50 Hz and 51–75 Hz ranges. In all other instances, the CCCs are all above 0.5, an indication that the regression analysis provided a satisfactory fit of the data.

Close inspection of Fig 1 suggests that there may have been a peak increase in power at the 6 μg/ml. This was most evident for the 30–50 Hz and 51–75 Hz bands for both cortex and thalamus as well as in the 76–125 Hz band for the cortex. To determine if this increase was significant, power at the 6 μg/ml concentration was compared with the average of baseline and 12 μg/ml power. There were significant outcomes before correction multiple comparisons for the 30–50 Hz bands for both recording sites as well as for cortical power in the 51–75 Hz band (Table 2). However, no difference remained significant after comparison for multiple comparisons (Table 2).

**Table 2:** Test hypothesis of a peak of log power at 6 μg/ml: 6 μg/ml versus mean of baseline and 12 μg/ml (mean ± std).

	log power at 6 mg/ml	log power mean of baseline and 12 μg/ml	Difference	CI	t (df = 8)	p	p adj
Cortex							
30–50 Hz	-0.51±0.64	0.26±1.01	0.77	-1.35 to -0.19	-3.08	***0*.*0152***	0.0932
51–75 Hz	-1.80±0.61	-1.20±1.01	0.60	-1.11 to -0.09	-2.71	***0*.*0265***	0.1596
76–125 Hz	-3.29±0.60	-2.92±0.92	0.37	-0.85 to 0.10	-1.81	0.1016	0.4474
125–200 Hz	-4.91±0.66	-4.71±0.83	0.20	-0.60 to 0.19	-1.19	0.2684	0.8052
Thalamus							
30–50 Hz	-1.14±0.51	-0.50±0.96	0.64	-1.16 to -0.12	-2.83	***0*.*0222***	0.1332
51–75 Hz	-2.35±0.39	-2.10±0.78	0.25	-0.69 to 0.19	-1.29	0.2309	0.6930
76–125 Hz	-3.56±0.39	-3.48±0.66	0.08	-0.56 to 0.38	-0.38	0.7118	0.8364
125–200 Hz	-5.00±0.73	-5.04±0.60	0.04	-0.42 to 0.50	0.21	0.8363	0.8364

CI: 95% confidence interval of the difference pair-wise difference of log power; p adj: p values adjusted for multiple (8) comparisons with Hommel's; p values of 0.05 or less are in ***bold italics***.

**Fig 2:**
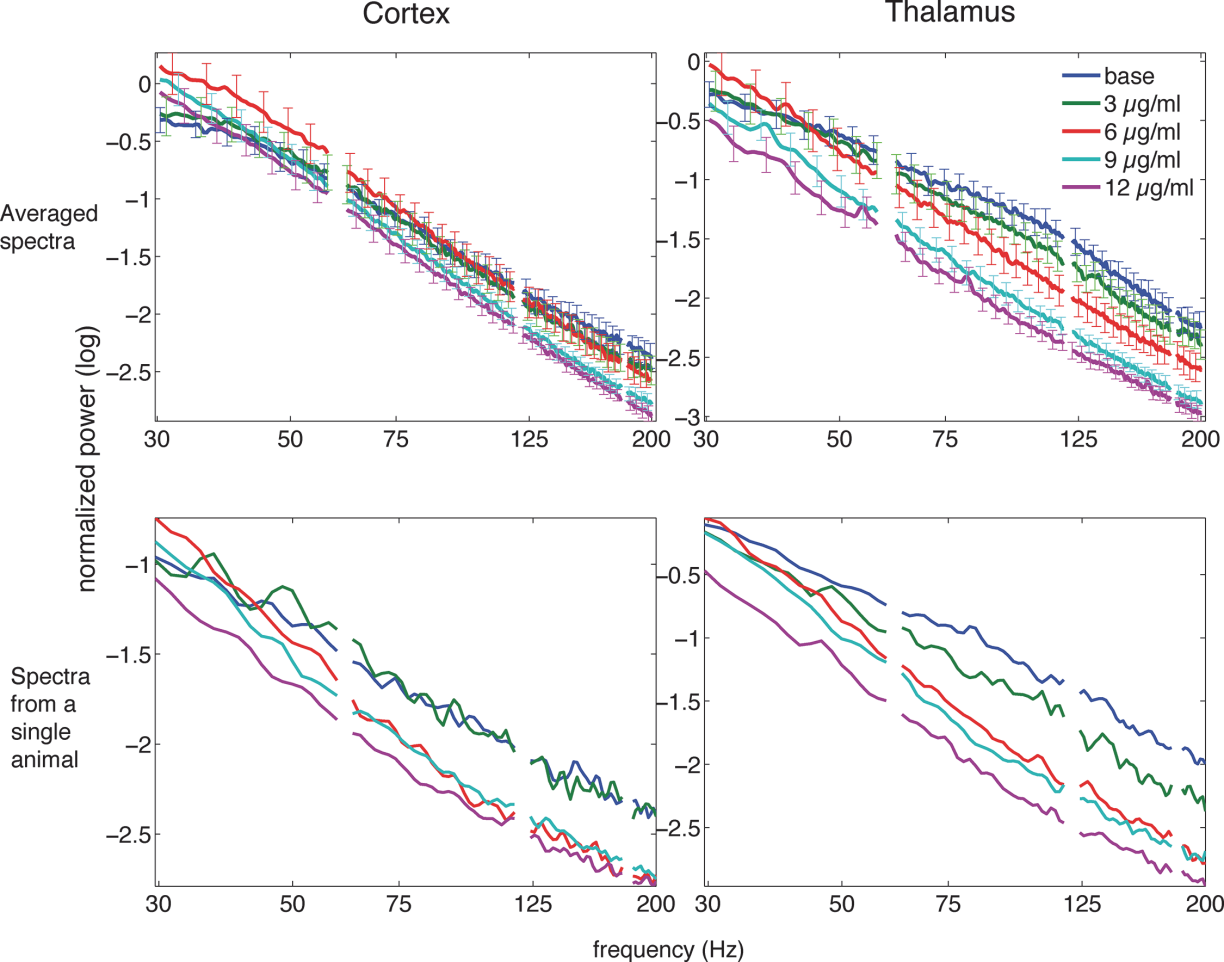
Power spectra (log-log scales). Top panel. Average spectra for all 9 animals for the cortex and thalamus. Bars show standard error. The blanks represent power excluded from analysis to minimize the impact of interference from external electrical sources, at 60, 120 and 180 Hz. Recovery data not shown to avoid distracting overlap. Bottom panel. Power spectra from one animal for the cortex and thalamus.

The concentration-dependent effect of propofol on the power of fast rhythms is readily noticeable on the average spectra with all animals combined (Fig 2 top panels) as well as on the power spectra from a single animal (Fig 2 bottom panels). The distance between the traces in these spectra is greater for the thalamus than for the cortex, an observation that is in line with the steeper negative slope of the concentration-effect plots for the thalamus.

**Fig 3:**
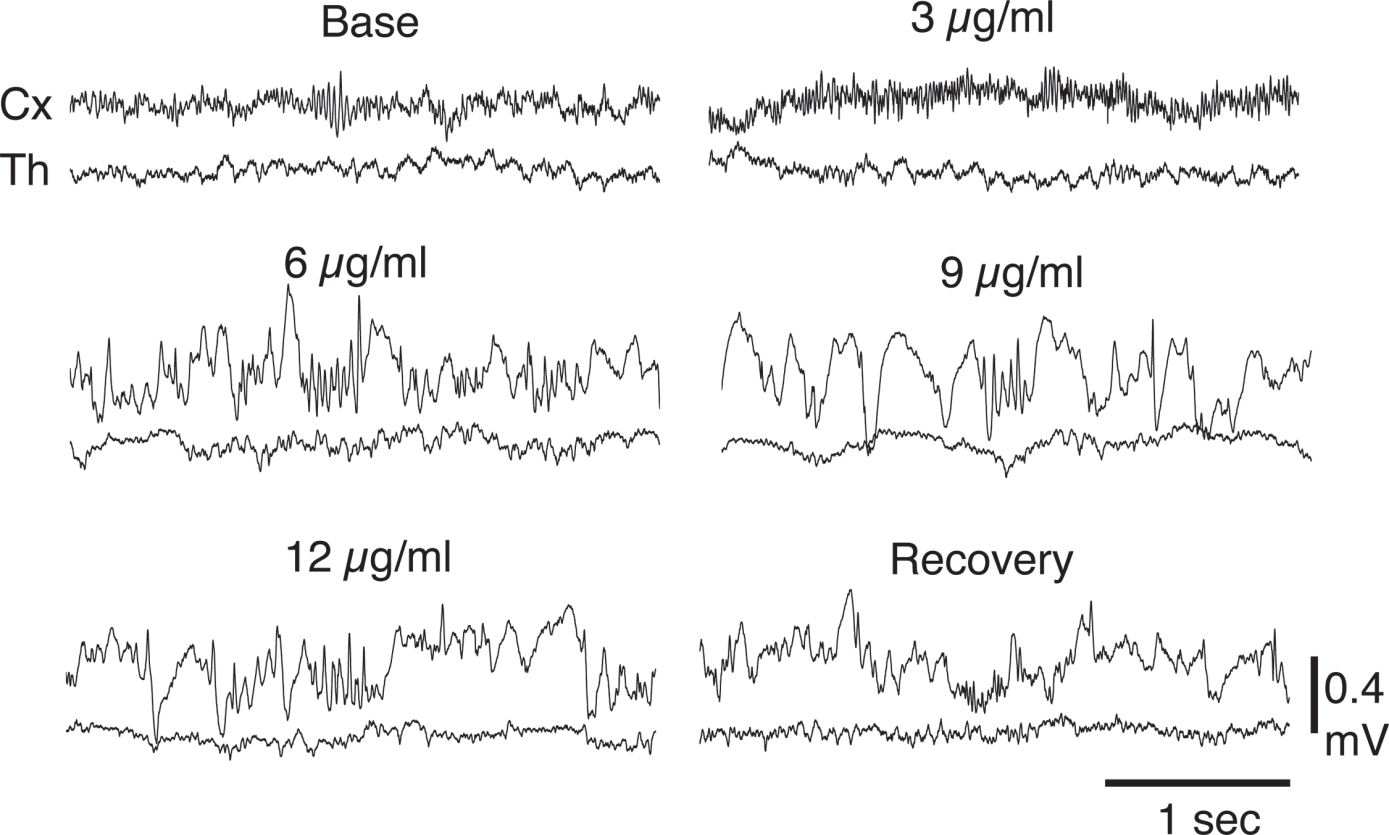
Segments of the original recordings from the same animal as Fig 2. The cortical traces show an increase in fast activity at the 3 μg/ml concentration. The 6, 9, and 12 μg/ml concentrations were associated with a progressive increase in slow activity and decrease of fast activity. The thalamic traces show minimal changes between baseline and the 3 μg/ml concentration. The 6, 9, and 12 μg/ml concentrations were associated with a progressive increase in slow activity and decrease of fast activity.

Segments of the original recordings from one animal are shown in Fig 3. The cortical traces show an increase in fast activity at the 3 μg/ml concentration. The other concentrations caused a progressive decrease of fast activity and a progressive increase in slow activity The thalamic traces show minimal changes between baseline and the 3 μg/ml concentration. The 6, 9, and 12 μg/ml concentrations were associated with a progressive increase in slow activity and decrease of fast activity.

### Relationship with level of consciousness

Our criterion for considering that the attenuation of power within a frequency band is related to the hypnotic effect is that the highest level of power within this frequency band observed during the periods when the animal is unconscious (i.e. the 9 and 12 μg/ml concentrations) should be less than the lowest level of power observed during any period when the animal is conscious. For cortical power, only the 126–200 Hz range satisfied this condition. Its highest value during unconsciousness (9 μg/ml concentration) was significantly less (p = 0.0476) than the lowest power during consciousness (recovery) (Table 3). For the 30–50 Hz range, highest cortical power during unconsciousness was unexpectedly significantly *higher* (p = 0.0160) than lowest power during consciousness (baseline). In the thalamus, highest power during unconsciousness (invariably at the 9 μg/ml concentration) was significantly reduced compared with the lowest power during consciousness (6 μg/ml concentration) for all frequency bands except the 30–50 Hz band (0.0112≤p≤0.0240) (Table 3).

**Table 3:** Power and level of consciousness.

	Periods	log power 1st period	log power 2nd period	Diff.	CI	t 8 df	p	p adj
Cortex								
30–50 Hz	base vs 9 μg/ml	-0.59±0.59	-0.19±0.70	-0.40	-0.63 to -0.18	-4.16	***#0*.*003***	***#0*.*016***
51–75 Hz	base vs 9 μg/ml	-1.63±0.67	-1.79±0.63	0.16	-0.19 to 0.53	1.07	0.316	0.316
76–125 Hz	reco vs 9 μg/ml	-3.14±0.63	-3.44±0.50	0.30	0.04 to 0.56	2.69	***0*.*027***	0.082
125–200 Hz	reco vs 9 μg/ml	-4.94±0.62	-5.23±0.52	0.29	0.08 to 0.50	3.24	***0*.*012***	***0*.*048***
Thalamus								
30–50 Hz	3 vs 9 μg/ml	-0.72±0.77	-1.16±0.68	0.44	-0.20 to 1.08	1.58	0.154	0.307
51–75 Hz	6 vs 9 μg/ml	-2.10±0.78	-2.79±0.45	0.69	0.28 to 1.10	3.87	***0*.*005***	***0*.*024***
76–125 Hz	6 vs 9 μg/ml	-3.48±0.66	-4.20±0.44	0.72	0.34 to 1.09	4.41	***0*.*002***	***0*.*014***
125–200 Hz	6 vs 9 μg/ml	-5.04±0.60	-5.66±0.61	0.62	0.31 to 0.92	4.65	***0*.*002***	***0*.*011***

Comparison of the period (baseline, 3 or 6 μg/ml concentrations or recovery) with the lowest mean level of power (estimated from the entire LFP epoch of 2 minutes and averaged across animals) observed when the animal is conscious with the concentration with highest mean spectral power observed during unconsciousness (9 μg/ml in all instances). mean±std

CI: 95% confidence interval of the difference pair-wise difference of log power; p adj: p values adjusted for multiple (8) comparisons with Hommel's; # Significant difference but in opposite direction (power during 9 μg/ml higher than during baseline); p values of 0.05 or less are in ***bold italics***.

### Recovery

The transition from the last concentration studied (12 μg/ml) to recovery was associated with a significant (0.0021≤p≤0.0162) increase in power in all instances (Table 4). Recovery recordings were obtained to confirm a return towards baseline power values to rule out other possible causes for the observed power changes (duration of experiment, equipment malfunction).

**Table 4:** To test the hypothesis that power during recovery is higher that during the 12 μg/ml concentration.

	log power at 12 μg/ml (mean±std)	log power recovery (mean±std)	Difference	CI	t 8 df	p	p adj
Cortex							
30–50 Hz	-0.43±0.76	0.11±0.69	0.54	-0.95 to -0.13	-3.03	***0*.*0162***	***0*.*0162***
51–75 Hz	-1.98±0.65	-1.43±0.73	0.55	-0.90 to -0.20	-3.59	***0*.*0071***	***0*.*0142***
76–125 Hz	-3.66±0.55	-3.14±0.63	0.52	-0.80 to -0.23	-4.16	***0*.*0032***	***0*.*0096***
125–200 Hz	-5.44±0.54	-4.94±0.62	0.50	-0.75 to -0.25	-4.66	***0*.*0016***	***0*.*0064***
Thalamus							
30–50 Hz	-1.58±0.72	-0.38±0.55	1.20	-1.78 to -0.60	-4.69	***0*.*0016***	***0*.*0064***
51–75 Hz	-3.07±0.58	-1.88±0.53	1.19	-1.77 to -0.62	-4.78	***0*.*0014***	***0*.*0056***
76–125 Hz	-4.55±0.53	-3.23±0.64	1.32	-1.83 to -0.82	-6.04	***0*.*0003***	***0*.*0021***
125–200 Hz	-5.94±0.76	-4.85±0.86	1.09	-1.51 to -0.65	-5.81	***0*.*0004***	***0*.*0028***

CI: 95% confidence interval of the pair-wise difference of log power; p adj: p values adjusted for multiple (8) comparisons with Hommel's procedure; p values of 0.05 or less are in ***bold italics***.

### Histology

**Fig 4:**
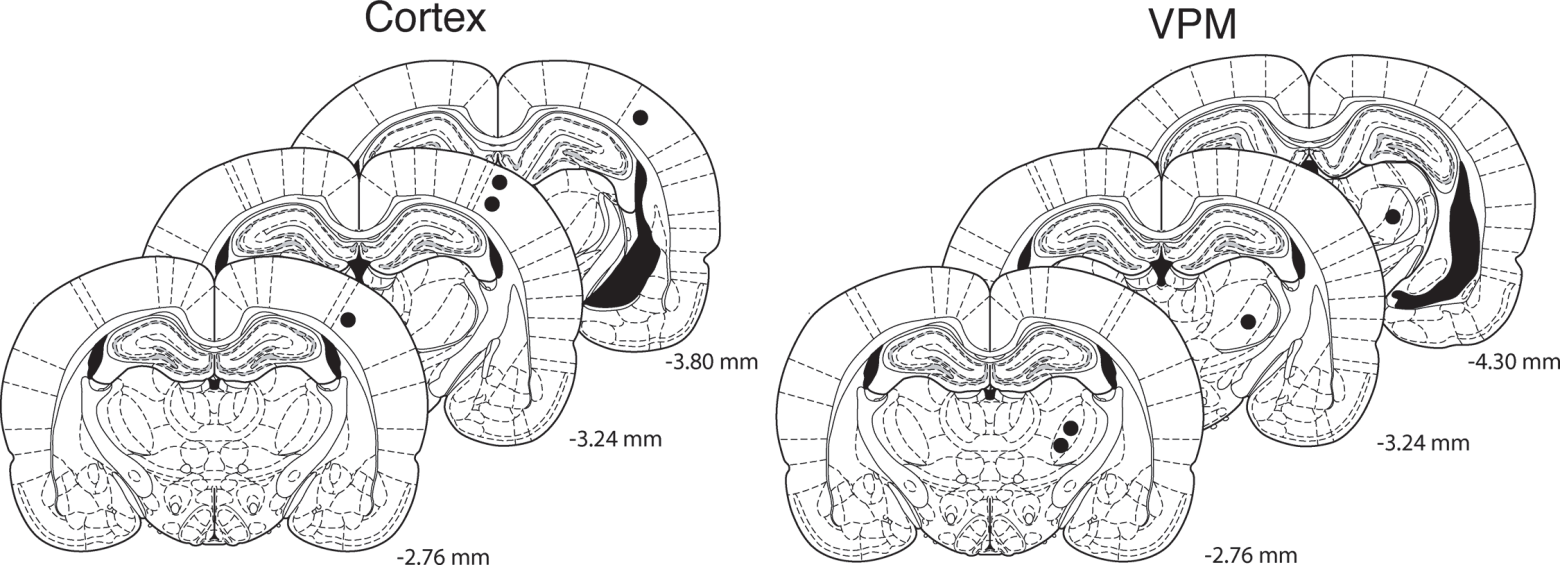
Histology. The filled circles denote the end of the electrode tracks on an overlay from the Paxinos atlas [22] and confirm correct electrode placement in the 4 available specimens. Distances are relative to bregma.

Accurate electrode placement was confirmed by microscopy in the four brains available (Fig 4).

## Discussion

The main finding of this study is that propofol causes a more extensive and pronounced attenuation of high-frequency rhythms in the sensory thalamus than in the sensory cortex. The attenuation of power of the high-frequency rhythms occurred over a wider frequency range in the thalamus (where all four frequency bands were affected) than in the cortex (where only the 76–125 Hz and 126–200 Hz bands were affected). More importantly, the slope of the concentration-effect line was steeper for the thalamus than for the cortex for all frequency bands and the differences were highly significant (p < 0.0018 for the 126–200 Hz range; p < 0.0001 for the other bands.

Another important finding of this study is that the attenuation of thalamic power appears to provide a more consistent marker of unconsciousness than cortical power. Thalamic power the 51–200 Hz range passed a fairly stringent test requiring that there be a significant difference between the highest level of power observed during the periods when the animal is unconscious (i.e. the 9 and 12 μg/ml concentrations) and the lowest level of power observed during the periods when the animal is conscious (baseline, propofol 3μg/ml or 6 μg/ml, recovery). For cortical power, only the 126–200 Hz range met these criteria. Further work on these rhythms appears warranted to assess their usefulness as potential markers of anesthetic-induced unconsciousness.

In regard to the issue of whether anesthetic-induced unconsciousness primarily results for impairment of the cortex, thalamus or both, [1] the present findings support the view that both structures are affected. This conclusion is in line with our recent report showing, on the basis of EEG recordings from the motor cortex and sensory thalamus in three patients, that induction of anesthesia with propofol is associated with concurrent alterations of cortical and thalamic activity [32].

Hudetz et al [12] had showed in rats that isoflurane attenuates spectral power in the 70–140 Hz range in neocortical and hippocampal recordings in a concentration-dependent manner. The present report confirms and extends these observations by showing that propofol exerts a similar effect on neocortical recordings. Breashears et al [14] had reported that propofol anesthesia is associated with a decrease of power in the 75–205 Hz range in human electrocorticograms. The present report shows that the same can be observed in an animal model. The importance of providing such confirmatory observations should not be underestimated. There is an increasing awareness of the need for replication in biomedical science in view of the alarmingly high proportion of findings that cannot be reproduced even though they were published in good faith by honest and competent investigators [33].

It is easy to forget that the results described above were obtained on data after logarithmic transform (base 10). Thus a significant linear term should not be interpreted as evidence that “power decreases linearly as a function of propofol concentration” but rather that “the logarithm of power decreases linearly as a function of propofol concentration”.

Our observation of a greater effect of propofol on the thalamus is in line the report of Ying et al [34]. These authors measured the concentration of propofol required to decrease synaptic responsiveness of thalamocortical relay neurons in the ventrobasal complex (VB) mouse brain slices and noted that this concentration was much less than for potentiation of GABA_A_ receptor-elicited responses in the hippocampus or olfactory cortex, suggesting that the ventro-basal thalamic neurons are more sensitive to propofol.

The attenuation of the power of high-frequency rhythms by propofol likely reflects a decrease of neuronal firing rates. Propofol decreases spontaneous neuronal firing rates in the both cortex [35, 36] and thalamus [35] by 30% or more and high-gamma power in LFPs is strongly correlated with the average firing rate[11, 37]. Concurrent recordings of both LFPs and of action potentials with the same electrode would allow verification of this interpretation.

Propofol exerted a puzzling effect on power in the 30–50 Hz range. It caused a concentration-dependent attenuation of thalamic, but not cortical, power in this range (Table 1). During unconsciousness, however, cortical power in the 30–50 Hz range was significantly *increased* compared with the period of consciousness with lowest power (Table 3). This is a clear indication that power in this band does not provide a good reflection of anesthetic effect, as noted by others [12]. The inconsistent effects of general anesthetics of the 30–50 Hz band may perhaps be justified to some extent by the fact that this frequency band occupies the border zone between the low frequency range where anesthetics increase power and the high-gamma range where anesthetics decrease power [38].

The inconsistent effects of propofol (and isoflurane [12]) on 30–50 Hz power are surprising given numerous observations that anesthesia in human subjects is associated with attenuation of “40 Hz” oscillations present in sensory evoked responses [39–41]. Furthermore, this effect is also concentration-dependent [3, 42, 43]. A key element to consider is that these studies did not look at spontaneous “40 Hz” activity but relied on the 40 Hz component of sensory evoked potentials [44]. The effects of isoflurane on these responses have been documented in the rat and are similar to those seen in humans [45].This confirms that there are important differences between spontaneous and evoked gamma oscillations [46].

### Limitations

We acknowledge two main limitations. First, we did not assay the propofol concentrations in blood to confirm the accuracy of the target controlled infusion protocol. We believe that collecting sequential arterial blood samples for each propofol concentration was not compatible with our testing protocol. Instead, we relied on predicted concentrations based on the Stanpump software and are thus dependent on the accuracy of the kinetic model [24]. In the worst-case scenario, we can conclude that we have demonstrated a dose-dependent (as opposed to concentration-dependent) effect since the dose of propofol and the rate of administration were similar across animals. While we are aware of prior use of target controlled infusion of propofol in rodents [47], we found no such studies where blood concentrations were measured. Second, histological confirmation of proper electrode placement is only available in four animals. Problems with tissue processing made the other specimens unusable. We nevertheless feel confident that the electrodes were properly positioned in all animals because we used electrophysiological guidance for fine adjustment of the antero-posterior position of the cortical electrode by measuring the cortical field excitatory post-synaptic potentials in response to electrical stimulation of the VPM.

### Conclusions

We conclude that propofol causes a concentration-dependent attenuation of the spectral power of thalamocortical rhythms in the 51–200 Hz range and that this attenuation is of sufficient magnitude to cause statistically significant reductions of power during unconsciousness. This effect is more consistent and more pronounced for the thalamus than for the cortex, suggesting greater thalamic sensitivity to propofol. Further work is warranted to determine to what extent and how this attenuation could contribute to the hypnotic action of propofol.

## Supporting Information

S1 Histology A histology slide showing the electrode tracks for cortex (matched to middle cut on Fig 4) and VPM (corresponding to the most posterior cut on Fig 4). (TIF)
