# Correction: Attenuation of High-Frequency (50-200 Hz) Thalamocortical EEG Rhythms by Propofol in Rats Is More Pronounced for the Thalamus than for the Cortex

**DOI:** 10.1371/journal.pone.0140087

**Published:** 2015-10-05

**Authors:** Sean J. Reed, Gilles Plourde

There is an error in the second sentence of the Results section in the Abstract. The correct sentence is: Propofol caused a robust linear concentration-dependent attenuation of cortical power in the 76–200 Hz range and of thalamic power in the 30–200 Hz range.

There is an error in the last sentence of the Results section in the Abstract. The correct sentence is: Furthermore the lowest concentration causing unconsciousness significantly reduced cortical power in the 126–200 Hz range and thalamic power in the 30–200 Hz range.

There is an error in the first sentence of the Conclusions section in the Abstract. The correct sentence is: Propofol causes a concentration-dependent attenuation of the power of thalamocortical rhythms in the 30–200 Hz range and this effect is far more pronounced for the thalamus, where the attenuation provides a robust correlate of the hypnotic action of propofol.
