# Supplementary figures and images for: Attenuation of High-Frequency (50-200 Hz) Thalamocortical EEG Rhythms by Propofol in Rats Is More Pronounced for the Thalamus than for the Cortex
Source: PLoS One. 2015 Apr 15;10(4):e0123287. doi: 10.1371/journal.pone.0123287 (PMC4398544; doi:10.1371/journal.pone.0123287)

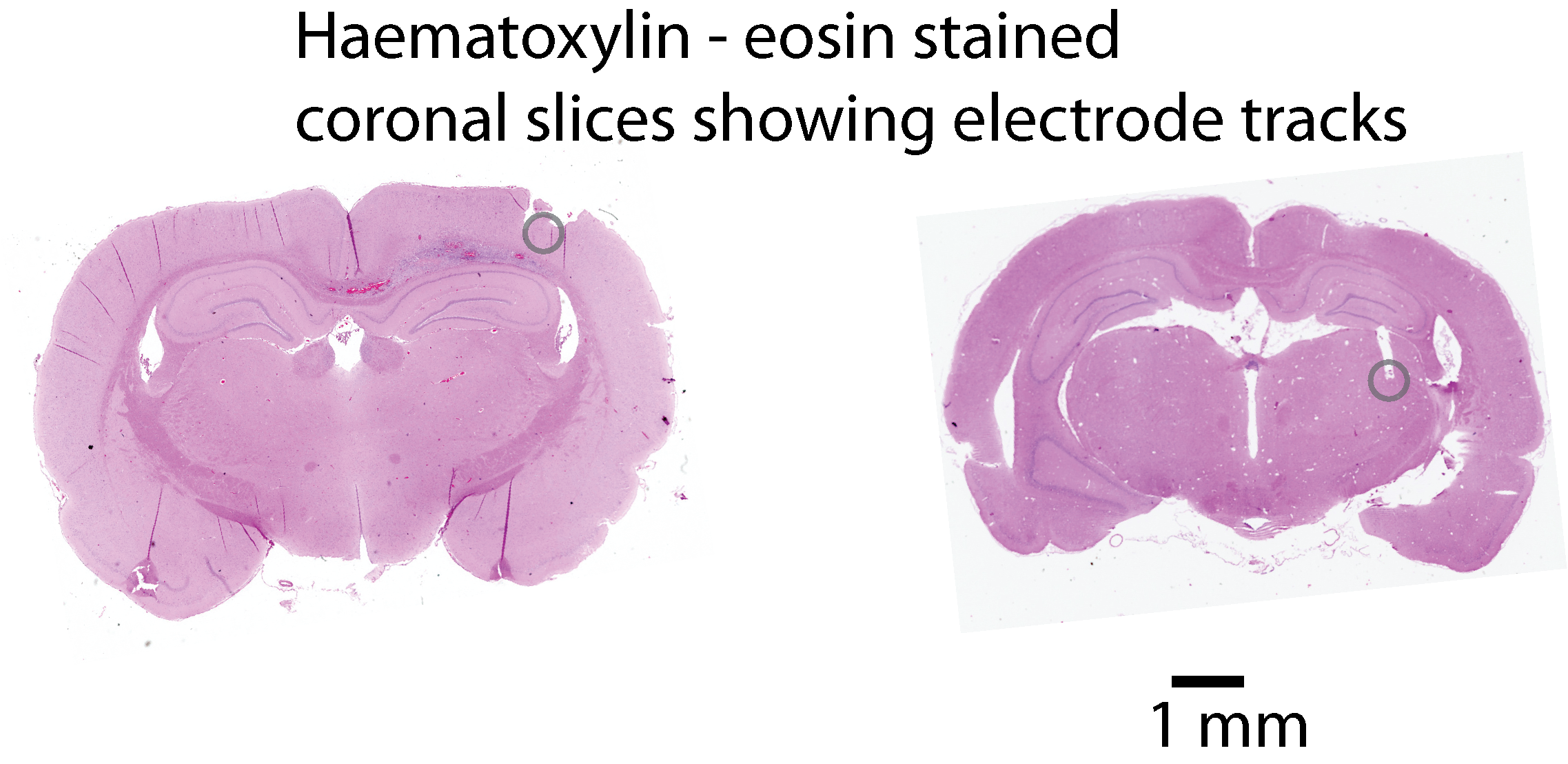

Supplement: S1 Histology — (TIF) [file pone.0123287.s001.tif]
